# Previously Undiagnosed Disease “X” in the Democratic Republic of the Congo: Malaria's Potential Role in the Outbreak

**DOI:** 10.1093/ofid/ofaf240

**Published:** 2025-04-18

**Authors:** Yura K Ko, Jesse Gitaka, Bernard N Kanoi, Billy E Ngasala, Mariko Kanamori, Wataru Kagaya, Akira Kaneko

**Affiliations:** Department of Microbiology, Tumor and Cell Biology (MTC), Karolinska Institutet, Solna, Sweden; Department of Virology, Tohoku University Graduate School of Medicine, Sendai, Japan; Center for Malaria Elimination, Institute of Tropical Medicine, Mount Kenya University, Thika, Kenya; Centre for Research in Infectious Diseases, Directorate of Research and Innovation, Mount Kenya University, Thika, Kenya; Center for Malaria Elimination, Institute of Tropical Medicine, Mount Kenya University, Thika, Kenya; Centre for Research in Infectious Diseases, Directorate of Research and Innovation, Mount Kenya University, Thika, Kenya; Department of Parasitology, School of Public Health, Muhimbili University of Health and Allied Sciences, Dar es Salaam, Tanzania; Department of Public Health Sciences, Stockholm University, Stockholm, Sweden; Institute for the Future of Human Society, Kyoto University, Kyoto, Japan; Department of Ecoepidemiology, Institute of Tropical Medicine (NEKKEN), Nagasaki University, Nagasaki, Japan; Department of Microbiology, Tumor and Cell Biology (MTC), Karolinska Institutet, Solna, Sweden; Department of Virology and Parasitology, Graduate School of Medicine, Osaka Metropolitan University, Osaka, Japan

To the editor—On November 29, 2024, the Democratic Republic of the Congo (DRC) alerted the World Health Organization (WHO) to an unusual increase in deaths in the Panzi health zone in Kwango Province. By December 5, 2024, 406 cases and 31 deaths were reported, with a concerning case fatality risk of 7.6%. Twenty-two (71%) of the fatalities occurred in individuals under age 15 years, with children <5 years accounting for 54.8%. Among 145 cases aged >15, 9 were fatal [1]. On December 17, 2024, the DRC's Ministry of Health announced that the disease in question was a severe form of malaria, and the WHO referred to the outbreak as acute respiratory infections complicated by malaria and acute malnutrition [2].

Given the suspicion that malaria significantly contributed to this outbreak, we analyzed historical malaria data to understand baseline risks and vulnerabilities in Kwango Province. We used data sets provided by Demographic Health Survey data (DHS) and the Malaria Indicator Surveys (MIS) across 20 Sub-Saharan African (SSA) countries [3]. For severe malaria, we applied the definition proposed by Taylor et al. in 2021 [4], based on signs and symptoms captured in DHS and MIS, which most closely align with the clinical symptoms of malaria outlined in the WHO Management of Severe Malaria Handbook. Loss of consciousness, rapid breathing, seizures, and severe anemia (hemoglobin levels <5 g/dL, adjusted for altitude) were defined as clinical manifestations of severe illness in children, consistent with the clinical features of severe malaria described in the WHO Handbook. The results showed that Kwango had moderate malaria prevalence (21.5%; 81/376) and high proportions of severe malaria cases (11.1%; 9/81) among children under 5 (Figure 1). Similarly, Malaria Atlas Project data indicate moderate to high *Plasmodium falciparum* prevalence and incidence in recent years (Supplementary Figure 1).

**Figure 1.**
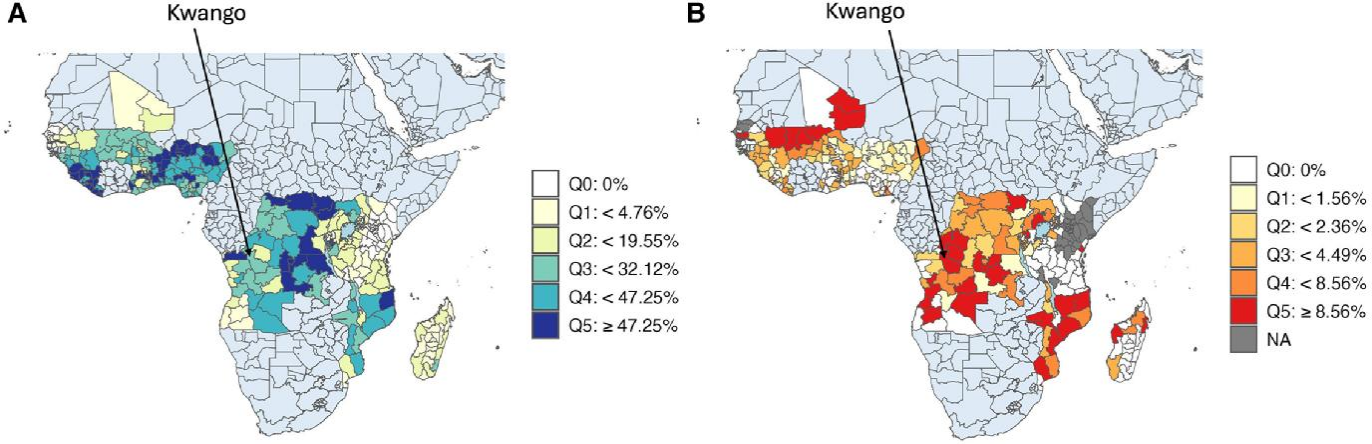
Proportion of (A) malaria-positive and (B) severe cases among malaria-positive children under 5 years old at Administrative Level 1 in 20 Sub-Saharan African countries: DHS data from 2014 to 2021. Kwango Province in the Democratic Republic of the Congo is indicated with the arrow. “NA” indicates areas with no malaria cases. Abbreviation: DHS, Demographic Health Survey.

Unlike typical epidemic-prone areas, such as highlands or semi-arid zones [5], where low malaria immunity triggers acute and severe outbreaks, Kwango's dense forests and low altitude (<1000 m) do not conform to this pattern. Indeed, Kwango has a history of continuous malaria transmission (Supplementary Figure 1). High mortality rates caused by malaria in such endemic areas, particularly among older children and adults, are unusual and warrant further investigation into malaria's role in the outbreak.

Several factors may contribute to the observed mortality trends. First, frequent malaria exposure might exacerbate other infections. Although the mechanisms by which malaria infection affects immune cells are not fully understood, the disease is known to cause immunosuppression lasting 2–8 weeks post-treatment [6]. Second, acute malnutrition can lead to treatment failures with artemisinin-based therapy [7]. Additionally, investigating whether the infecting parasites exhibit partial resistance to artemisinin is crucial. In Uganda, which borders the DRC, delayed response to artemisinin in severe malaria cases among children was reported [8]. The epidemic may be influenced by rising temperatures and more variable precipitation, which are pressing issues in the province [9]. Moreover, limited health care access, shifts in vector populations, and population movements may compound malaria's impact on the outbreak.

However, it is also essential to recognize that the presence of *Plasmodium* parasites or their antigenic proteins in patients does not necessarily indicate that malaria is the cause of illness. Dalrymple et al. reported that less than one-third of all fevers in *P. falciparum*–infected children under 5 were causally attributable to *P. falciparum* [10].

While our letter focuses specifically on the role of malaria and does not comprehensively address other possible disease causes, future outbreak investigations should refrain from prematurely attributing an epidemic to malaria based solely on malaria diagnostic test results.

## Supplementary Material

ofaf240_Supplementary_Data

## Supplementary Data

Supplementary materials are available at *Open Forum Infectious Diseases* online. Consisting of data provided by the authors to benefit the reader, the posted materials are not copyedited and are the sole responsibility of the authors, so questions or comments should be addressed to the corresponding author.
