# Supplementary material for: Previously Undiagnosed Disease “X” in the Democratic Republic of the Congo: Malaria's Potential Role in the Outbreak
Source: Open Forum Infect Dis. 2025 Apr 18;12(5):ofaf240. doi: 10.1093/ofid/ofaf240 (PMC12063206; doi:10.1093/ofid/ofaf240)
Supplement: ofaf240_Supplementary_Data [file ofaf240_supplementary_data.pdf]

Supplementary Materials: Previously undiagnosed disease "X" in the Democratic Republic of the Congo: malaria's potential role in the outbreak

Authors:
Yura K Ko[1,2], Jesse Gitaka[3,4], Bernard N. Kanoi[3,4], Billy E. Ngasala[5], Mariko Kanamori[6,7], Wataru Kagaya[8], Akira Kaneko[1,9]

Institutions of affiliation:
[1]Department of Microbiology, Tumor and Cell Biology (MTC), Karolinska Institutet, Sweden
[2]Department of Virology, Tohoku University Graduate School of Medicine, Japan
[3]Center for Malaria Elimination, Institute of Tropical Medicine, Mount Kenya University, Kenya
[4]Centre for Research in Infectious Diseases, Directorate of Research and Innovation, Mount Kenya University, Kenya
[5]Department of Parasitology, School of Public Health, Muhimbili University of Health and Allied Sciences, Tanzania
[6]Department of Public Health Sciences, Stockholm University, Sweden
[7]Institute for the Future of Human Society, Kyoto University, Japan
[8]Department of Ecoepidemiology, Institute of Tropical Medicine (NEKKEN), Nagasaki University, Japan
[9]Department of Virology and Parasitology, Graduate School of Medicine, Osaka Metropolitan University, Japan

Data sources

We used datasets provided by the Demographic Health Surveys (DHS) program [1] and the Malaria Indicator Surveys (MIS), with no direct contact with the study participants. Informed consent was not required for this analysis, as it relied on secondary data without participant interaction. The DHS program adheres to established international and local ethical standards and protocols in its surveys. Details of the specific datasets used for each country are provided in Table S1.

Table S1: Year and data source for the analysis

Country	Data
Angola	DHS 2015-2016
Benin	DHS 2017-2018
Burkina Faso	MIS 2017-2018
Burundi	DHS 2016-2017
Democratic Republic of the Congo	DHS 2014
Ghana	MIS 2019
Guinea	MIS 2021
Kenya	MIS 2020
Liberia	MIS 2016
Malawi	MIS 2017
Mali	DHS 2018
Nigeria	DHS 2018
Madagascar	MIS 2016
Mozanbique	MIS 2018
Rwanda	DHS 2019-2020
Senegal	DHS 2017
Sierra Leone	MIS 2016
Tanzania	MIS 2017
Togo	MIS 2017
Uganda	DHS 2018-2019

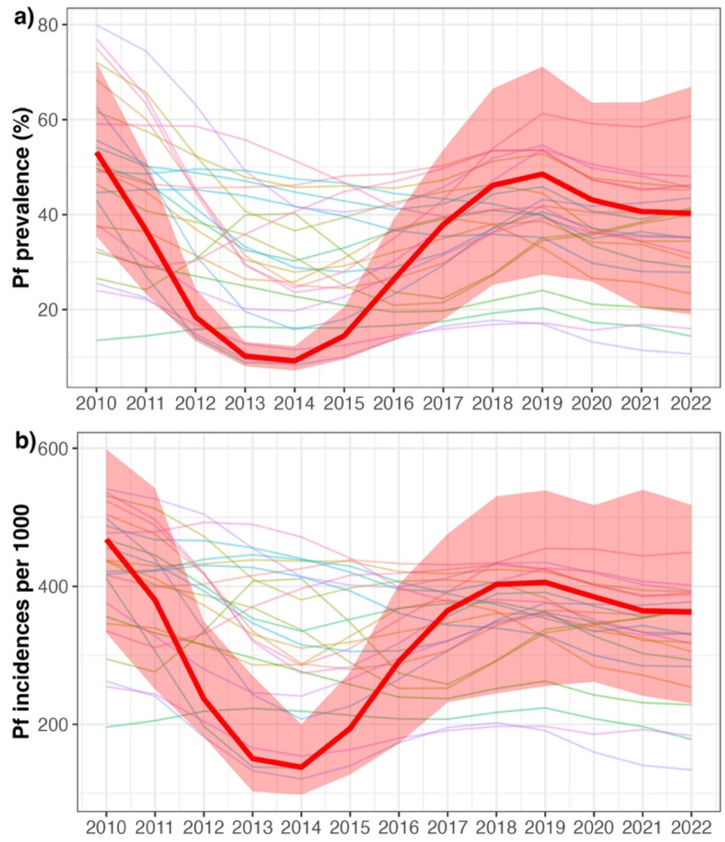

Figure S1. ***Plasmodium falciparum*** **a) prevalence and b) annual incidence in 26 provinces in the Democratic Republic of the Congo (DRC). The red line shows Kwango province, and the shaded area shows the 95% credible interval: Malaria Atlas Project data** [2]

References

[1] “The Demographic Health Surveys.” [Online]. Available: https://dhsprogram.com/.
[2] S. I. Hay and R. W. Snow, “The malaria Atlas Project: developing global maps of malaria risk,” *PLoS Med.*, vol. 3, no. 12, p. e473, Dec. 2006.
